# Novel Electrode Designs for Neurostimulation in Regenerative Medicine: Activation of Stem Cells

**DOI:** 10.1089/bioe.2020.0034

**Published:** 2020-12-16

**Authors:** Stephanie N. Iwasa, HaoTian H. Shi, Sung Hwa Hong, Tianhao Chen, Melissa Marquez-Chin, Christian Iorio-Morin, Suneil K. Kalia, Milos R. Popovic, Hani E. Naguib, Cindi M. Morshead

**Affiliations:** ^1^ The KITE Research Institute, Toronto Rehabilitation Institute—University Health Network, Toronto, Canada.; ^2^ CRANIA, University Health Network and University of Toronto, Toronto, Canada.; ^3^ Department of Mechanical and Industrial Engineering, University of Toronto, Toronto, Canada.; ^4^ Department of Chemical Engineering and Applied Chemistry, University of Toronto, Toronto, Canada.; ^5^ Institute of Biomedical Engineering, University of Toronto, Toronto, Canada.; ^6^ Department of Neurosurgery, University Health Network, University of Toronto, Toronto, Canada.; ^7^ Krembil Research Institute, Toronto, Canada.; ^8^ Department of Materials Science & Engineering, University of Toronto, Toronto, Canada.; ^9^ Department of Surgery, University of Toronto, Toronto, Canada.

**Keywords:** electrical stimulation, cell migration, electrode design

## Abstract

Neural stem and progenitor cells (i.e., neural precursors) are found within specific regions in the central nervous system and have great regenerative capacity. These cells are electrosensitive and their behavior can be regulated by the presence of electric fields (EFs). Electrical stimulation is currently used to treat neurological disorders in a clinical setting. Herein we propose that electrical stimulation can be used to enhance neural repair by regulating neural precursor cell (NPC) kinetics and promoting their migration to sites of injury or disease. We discuss how intrinsic and extrinsic factors can affect NPC migration in the presence of an EF and how this impacts electrode design with the goal of enhancing tissue regeneration. We conclude with an outlook on future clinical applications of electrical stimulation and highlight technological advances that would greatly support these applications.

## Introduction

Neurological disorders, including stroke, Alzheimer's, and Parkinson's disease, are the leading cause of disability and the second leading cause of death globally.^1^ Recent advancements in bioelectricity research, conductive polymers, and carbon-based materials have the field poised to treat these neurological disorders using electrical stimulation by way of enhancing endogenous neural repair. The opportunity is afforded by the presence of electrosensitive resident neural stem and progenitor cells (termed neural precursor cells, NPCs) in the brain and the innovative approaches underlying novel electrode designs. New materials with improved mechanical, electrical, and chemical properties, including greater flexibility, conductivity, and biocompatibility, provide researchers with new options to implant and deliver electrical stimulation and promote neural repair.

Bioelectricity was discovered over 200 years ago and, since then, researchers have discovered that endogenous electric fields (EFs) are vital for proper development and wound healing. Disruption or reversal of these fields can cause developmental deformations and prevent tissue repair.^2,3^ Decades of investigation have examined the role of electrical stimulation in enhancing wound healing, particularly for skin and bone in animal models and clinical trials.^4–6^ The field of bioelectricity has highlighted our understanding of the diverse and profound responses of cells to EF application upon which to build our regenerative strategies.

Technology in the 1960's and 1970's focused on implanting devices into the central nervous system (CNS) to deliver electrical stimulation specifically for pain.^7^ Now, deep brain stimulation (DBS), spinal cord stimulation, peripheral nerve stimulation, vagus nerve stimulation, transcranial magnetic stimulation, and functional electrical stimulation are all examples of the successful application of clinical electrical stimulation to benefit patients.^8–13^ These techniques are widely available globally, with well-measured clinical outcomes, and in some cases are now standard of care. While the outcomes are well understood, the mechanisms underlying the success of neuromodulation therapies are less well-defined, although modification of neural circuits and action potential-generating cells has been shown to result from these interventions. DBS is standard of care for the treatment of appropriately selected patients in movement disorders and epilepsy and may be an option for patients with certain types of pain syndromes.^9^ Spinal cord stimulation has been used for decades to reduce chronic neuropathic pain.^8^ More recently, transcranial magnetic stimulation has been approved to treat depression^12^; and functional electrical stimulation has been used for decades to restore motor and sensory functions following CNS injury.^13^ Another treatment that uses electrical stimulation is tumor treating fields. These EFs do not focus on neuroplasticity or modifying neural circuits but instead focus on disrupting tumor cell mitosis through high-frequency electrical stimulation. Tumor treating fields were FDA approved in 2011 to treat glioblastoma multiforms.^14^ These varied uses with considerable success demonstrate the versatility of treatments using electrical stimulation.

We hypothesize that neural repair ensues upon application of electrical stimulation as a result of EF generation that modulates the behavior of nonaction potential-generating cells. This could include glial cells and vascular endothelial cells but most promising is the activation of electrosensitive resident NPCs. It has been demonstrated that NPCs are highly responsive to EF application and are activated to proliferate, differentiate, and migrate in response to EF application.^15^ Migration due to EFs has been extensively demonstrated *in vitro*, and more recent studies show the ability of applied EFs to promote NPC migration along migratory paths *in vivo* in the rodent brain.^15–18^ Differentiation and proliferation kinetics can also be modified by electrical stimulation, and this has been demonstrated both *in vitro* and *in vivo*.^19–21^ Optimization of these EFs to better control NPC behavior is still required, but manipulating NPC behavior affords great promise in the field of regenerative medicine.

As we consider the goal of developing novel therapeutics for brain repair, herein we will discuss the cellular outcomes following EF application and ongoing work designed to fully understand the response of CNS tissue to EFs. We will consider not only the ways to maximize the cell-based response (from genes to migration) but also importantly we will consider the optimization of EF-based activation strategies and how cellular outcomes will feed into the design elements of the electrodes. We will highlight the response of resident NPCs to EF application in terms of survival and neurogenesis and focus more specifically on EF-induced migration (galvanotaxis), as this is a critical step to ensuring that sufficient numbers of cells are available to contribute to neural repair. The various electrode materials and geometry design for optimizing galvanotaxis will be discussed in detail. Finally, we will conclude with some exciting potential clinical applications and technological advances.

## What Influences Galvanotaxis? Nature Versus Nurture

Endogenous NPCs are rare, comprising less than 10% of the periventricular cells in a three to five cell layer thick region lining the lateral ventricles in the adult forebrain. These NPCs are highly responsive to EF application, expanding in number through proliferation and enhanced cell survival, as well as differentiating into newborn neurons.^19^ Most striking, NPCs are activated to migrate in a rapid and directed manner in the presence of an applied EF using well-established *in vitro* assays and live cell imaging.^15^ Together, these NPC behaviors provide promise for the design and implementation of regenerative medicine strategies that aim to replace lost or damaged cells following injury or disease, yet several important questions remain unanswered that are pivotal to understanding how to optimize the NPC response. For instance, how does a cell sense the EF? What is the intracellular signaling cascade(s) that dictates the EF-induced cell behavior? Indeed, electrosensitive cells can differ in their migratory response to the same EF application by migrating in different directions (cathodal vs. anodal), with different speeds and distinct migratory pathways (tortuosity). These behaviors are not only cell-dependent but also regulated by the stimulation paradigm and the microenvironment. Considering these factors together, one can envision that EF application would lead to a highly interactive, niche-dependent cellular response in injured or diseased tissue.

### Nature: intrinsic cell migration mechanisms and responses to stimulation

The same EF application can lead to specific responses in distinct cell populations.^22^ Indeed, cells display directedness in an EF, migrating toward the cathode (negative) or anode (positive) depending on the cell type. What mechanisms may underlie these different responses? For a cell to start migrating in one direction, the cell needs to first sense the EF which will ultimately lead to asymmetry within the cell through signaling cascades that enhance migration in one direction (e.g., extension of the cytoskeleton). This galvanotactic response can involve the electrophoresis of charged membrane proteins following electrical stimulation, which creates a ligand gradient along the cell membrane, thereby generating asymmetry within the cell.^23^ Another response to the EF is the polarization of charged molecules within the cell, which can lead to asymmetry by binding and blocking channels on the cell surface.^22^

An equally plausible hypothesis is the presence of multiple EF-sensing mechanisms and signaling cascades that could, in theory, underlie migration in opposite directions from a resulting “tug-of-war” between mechanisms within a single cell. An example of a pathway involved in translating EF signals into migration is the phosphoinositol-3 kinase (PI3K) pathway. PI3K is a central enzyme involved in the signal transduction of stimuli, including growth factors and cytokines. Blocking PI3K significantly decreases galvanotactic response in many cell populations suggesting its important role in sensing the EF.^16,24–26^ Furthermore, studies have demonstrated that blocking guanylyl cyclase, an enzyme involved in the signal transduction of many cell processes like proliferation and migration, can completely reverse the direction of migration resulting in a cathodally-migrating cell becoming an anodally-migrating cell.^25^ Dissociating the speed of migration and the direction of migration highlights the complexity of the response and the presence of more than one signaling cascade underlying the galvanotactic response.

In general, increasing EF strength results in a graded increase in speed of migration until the cells undergo cell death from the high EF strength. Human NPCs will migrate in a directed manner in an EF strength of 250–350 mV/mm and undergo rapid cell death in higher EF strengths.^27^ Most interesting, the species from which the NPCs are derived can influence their migratory response. For instance, mouse-derived NPCs migrate to the cathode, while human-derived NPCs migrate to the anode in the same EF strength and when placed on the same substrate.^15,27^ NPCs derived from human embryonic stem cells or directly reprogrammed from mature human bone marrow can migrate toward the cathode in the presence of an EF.^18,27^ These findings suggest that galvanotaxis is a common feature of NPCs, irrespective of the origin of the cells. Bovine-derived epithelial cells are another example of a cell population that undergoes galvanotaxis but the direction of migration is dependent on the strength of the applied EF. These studies highlight the fact that different mechanisms appear to underlie EF induced migration of distinct cell populations.^28^ When considering *in vivo* application, it is important to consider that the vast majority of *in vitro* studies use direct current electrical stimulation. The use of direct current electrical stimulation requires that the electrodes and cells be placed in separate chambers to prevent toxic by-products generated from the electrode–electrolyte interface from influencing the cells.

For *in vivo* application, toxic by-products at the interface are reduced by stimulating with a charge-balanced pulse (i.e., the amount of charge injected into the tissue will equal the amount of charge drawn out of the tissue). Toward the goal of *in vivo* application, the effects of charge-balanced biphasic monopolar stimulation on NPC migration *in vitro* were examined and it was found that the frequency was a key element of NPC galvanotaxis.^29^ Higher frequencies were effective in promoting migration, whereas lower frequencies were not. We postulate that the increased time between pulses (lower frequency) allows the polarized or electrophoresed membrane proteins to move back to baseline conditions eliminating the asymmetrical activation of intracellular signaling cascades required for enhancing migration in one direction.

Biphasic stimulation is yet to be optimized *in vitro*, as is the applied electrical stimulation required to deliver EF strengths *in vivo* to promote galvanotaxis. However, promising recent work has demonstrated that biphasic *in vivo* stimulation can enhance NPC migration and regulate cell behavior.^17,19^ Transferring the *in vitro* parameters to *in vivo* settings will be a challenge as the microenvironment also has profound effects on galvanotaxis. The microenvironment plays an important role in what the cell perceives and as such, the galvanotactic response is highly sensitive, yet malleable, with the outcome dependent on the EF parameters such as strength and frequency and the cell's environment (e.g., extracellular matrix [ECM] and nearest neighbors, discussed next).

### Nurture: extrinsic microenvironment factors influencing migration responses

**FIG. 1.**
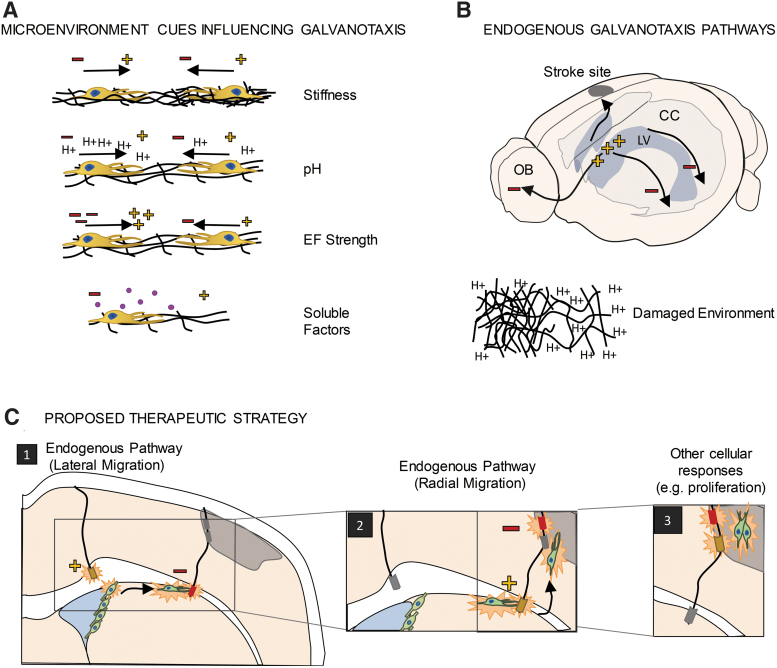
Galvanotaxis. **(A)** Differences in the microenvironment can affect the galvanotactic response, including reversing the direction and speed of galvanotaxis. **(B)** Endogenous electric potential differences are found *in vivo* and these are consistent with migration pathways found *in vivo*. Damaged tissue will have different microenvironments which could either affect galvanotactic response or serve as a migratory cue. Harnessing these pathways could facilitate electrical stimulation therapies. **(C)** Proposed therapeutic strategy utilizing endogenous pathways and fine-tuning the electrical stimulation to elicit different cellular responses. Minus (−), cathode; plus (+), anode; CC, corpus callosum; EF, electric field; LV, lateral ventricle; OB, olfactory bulb.

The microenvironment is a complex combination of cues, which include cell–cell interactions and interactions with extracellular matrices and soluble and tethered physical factors (Fig. 1A). Different microenvironments are found during aging, disease, and injury, including altered extracellular membrane proteins, changes in pH, the presence of infiltrating blood cells, and changes in neighboring cell phenotypes (e.g., formation of a glial scar by activated astrocytes; activation of microglia which are the resident immune cells in the CNS), which ultimately alter cell behavior in response to electrical stimulation.^15,30,31^ For example, after injury, the ECM becomes more flexible, the pH is reduced, and pro-inflammatory factors are expressed near the injury site.^32–34^ These are all factors which can affect cell migration. It is important to understand how different brain microenvironments may affect the efficacy of galvanotaxis in a clinical setting and further to consider how altering the microenvironment through implanted electrodes may impact the galvanotactic response.

Altered levels of mitogens or increased cytokine release are good examples of factors that are affected by injury or disease and can impact NPC migration.^35^ For instance, the mitogen EGF is critical for the rapid and cathodally-directed galvanotaxis of murine NPCs such that blocking EGF signaling leads to slower cell migration, with no change in directionality.^15^ After ischemic injury, EGF is upregulated in damaged tissue, as well as the NPC niches in the brain.^36^ Injuries can also activate and recruit inflammatory cells leading to the release of cytokines which alter calcium and pH levels which are known to impact galvanotaxis.^37,38^ Indeed, extracellular pH can completely reverse the direction of migration (i.e., from cathodal to anodal) in keratinocytes.^30^ This is thought to be due to changes in ion channel activity such as potassium channel Kir4.2, which has been shown to be instrumental in sensing EFs. Hence, the regulation of the galvanotactic response is highly sensitive to the microenvironment, and the different migratory parameters (speed, direction) can be independently regulated by specific cues. Innovations in electrode design could include the delivery of molecules to regulate the microenvironment to control galvanotaxis.

Perhaps most compelling is some recent work highlighting the role of the ECM in galvanotaxis. Ahmed et al., studied human derived NPCs in the presence of an applied EF and reported that substrate stiffness was sufficient to completely reverse the direction of migration.^27^ Whether the substrate was a cell monolayer or fibrous protein, the direction of migration of human NPCs was dictated by the stiffness of the substrate, while the speed was unaffected. Considering the physical properties of the different regions of the brain (i.e., white matter axon tracts vs. gray matter neuronal cell bodies), as well as the scar formation after injury (composed of activated glial cells, which are less stiff than uninjured brain tissue), the impact on NPC based neuroplasticity is significant.

Another consideration for the development of electrical stimulation therapy is the endogenous EFs present within the tissue (Fig. 1B). Indeed, in the mature CNS endogenous EFs have been shown to play a role in NPC migration under baseline conditions.^17,39^ An endogenous EF exists along the rostral–caudal axis, which is a pathway for NPC to migrate to the olfactory bulb where they generate new olfactory bulb interneurons throughout life. The small endogenous EF (∼3 mV/mm) is thought to be the result of ion distribution in the extracellular space and differential ion pump distribution on the apical and basal surface of epithelial cells comprising the NPC niche.^39^ Reversing this endogenous EF causes cells to migrate in the opposite direction along this same rostro-caudal axis, supporting its role in migration.^18,39^

More recently, an electric potential difference was identified in the mature CNS along the medial-lateral axis, specifically along the corpus callosum (the largest white matter tract in the forebrain). This endogenous EF was coincident with the lateral migration of transplanted NPCs on the corpus callosum and, again, reversing the electric potential resulted in NPC migration in the opposite direction.^17^ Hence, enhancing cell migration to a site of injury or disease will also need to consider the presence of endogenous EFs that persist, or are generated, in response to injury, and may need to be overcome to enhance targeted migration.^40,41^

Currently, there are limited *in vivo* studies that have investigated transplanted NPC galvanotaxis in the rodent brain along these migratory paths.^17,18^ In these studies, fluorescent NPCs were visualized through immunohistochemistry, which provided snapshots of their migration. The respective electrical stimulation paradigms revealed migration ranging from ∼100 μm over 3 days to as much as 6 mm over the course of months. Details regarding migration path and speed in the brain's three-dimensional (3D) microenvironment were not determined as this was a limitation of using immunohistochemistry at single time points to evaluate the cellular response. Next steps to acquire higher spatial and temporal resolution will provide insight into these important aspects of *in vivo* galvanotaxis.

Nevertheless, together, these studies support the hypothesis that exogenous application of EFs will provide cues that can regulate NPC behavior and support neural repair (Fig. 1C). Notably, electrical stimulation can elicit other cellular responses, such as cell proliferation.^19^ Interestingly, electrical stimulation has been shown to increase the number of blood vessels in the injured brain,^42^ modulate blood–brain barrier permeability,^43,44^ and modulate numbers of microglia and astrocytes.^45–48^ The ability to affect the microenvironment creates the possibility of “side effects” such as modulation of the number of astrocytes and microglia but with more insight into the effects of EFs on tissue responses; these “side effects” could be purposely controlled to create an environment more amenable to tissue repair.^45–48^ Development of this therapy requires exquisite attention to design parameters to manufacture novel electrodes that will function in a range of microenvironments to facilitate the desired galvanotaxis response.

## What Do You Need in an Electrode? Stimulation, Flexibility, and Compatibility

Design of electrodes for producing the EFs to augment NPC behaviors for neural repair requires the consideration of a number of factors. Current electrodes are rigid, and the implantation can serve as a source of tissue injury, ultimately impacting NPC migration, as described. Therefore, to successfully deliver this therapy, it is important to develop flexible electrodes with novel biocompatible materials that can be tuned to deliver appropriate electrical stimulation. Considerations such as electrode geometry can also be refined to provide additional customizable parameters depending on the location of the implants and the age, injury, or disease state of patients in need of neural repair.

### Electrical stimulation: how shocking is it?

Characterization of the electrical properties of the electrode and the tissue is required to predict what EF cells are experiencing and to predict the outcomes. An important parameter is impedance, of both the electrode and the tissue. Impedance is the frequency-dependent current-voltage response that describes the dynamic electrical properties of a system. It is commonly defined as the opposition to alternating current and has two components: resistance and reactance. Resistance is the frequency-independent opposition to current, while reactance is the frequency-dependent combination of capacitance and inductance that oppose alternating current.^49,50^

The most common techniques used to characterize the electrical properties of an electrode are electrochemical impedance spectroscopy (EIS) and cyclic voltammetry (CV). EIS measures electrical impedance for a wide range of different frequencies, while CV measures current density for a range of potentials. These can be tailored to values that elicit a biological response and used to characterize the electrode and the electrode-tissue interfacial properties, which are critical for *in vivo* application. The results can also be derived through an electrical model that represents the electrode as an equivalent circuit and are dependent on the stimulus parameters such as pulse amplitude, frequency, and pulse duration.^51,52^ For neural stimulation, a biphasic electrical stimulation is typically applied to prevent charge accumulation, which is associated with pH changes and overpotential.^53^ Significantly, it is only of late that charge-balanced electrical stimulation was shown to induce NPC migration *in vitro* and now *in vivo.*^17,29^ This is an exciting and positive step when considering EF application for NPC-based neural repair strategies.

Monitoring impedance is an important way to determine the efficacy of implanted electrodes as the degree of impedance (i.e., too large or too small) can indicate problems with the design or equipment.^54^ In general, for implanted stimulating electrodes, high current densities while operating are usually required and, as such, benefit from lower impedances.^55^ The impedance will vary depending on the stimulation parameters, the tissue environmental parameters such as temperature, and the electrode's material, surface area, and geometry.^56^ Even different regions of the brain, white matter, gray matter, and cerebral spinal fluid, have different electrical impedances which can further change through aging and disease. Indeed, the time of implantation relative to stimulation can also affect the impedance observed^51,52,56–58^; thus, it is critical to generate a comprehensive model to provide a clear understanding of the EF perceived by the NPCs in an applied EF.

### Be flexible and biocompatible: fitting in

Materials such as platinum and its alloys, iridium oxide and titanium nitride, have been used extensively for creating implantable stimulation electrodes for the nervous system due to their low impedance and biocompatibility. These conventional metal-based electrodes have been reviewed previously, detailing information on electrode performance and potential drawbacks due to electrode degradation.^59^

One of the features of these currently used electrodes is their inflexible nature and the damage that can ensue following implantation, including mechanical disruption and tissue inflammation, ultimately resulting in changes to the microenvironment that can alter NPC behavior. To reduce the perturbation to the microenvironment, a biocompatible material that closely matches the stiffness of the brain would be ideal. Toward this end, a set of novel materials, including conducting polymers and carbon-based nanoparticles, have been used for adaptation as neural stimulation electrodes. These novel electrode materials afford benefits such as high charge injection density, high electrical conductivity, high flexibility, low toxicity, and electrical tunability. Most of these materials have been tested *in vitro* for biocompatibility and some have been tested for their ability to elicit an NPC behavioral response.^60–62^ Materials discussed are summarized in Table 1.

**Table 1.** A Review of Materials for Neural Electrical Stimulation, Their Properties, Advantages, and Disadvantages

Electrode materials	Fabrication process	Geometry	Electrical resistivity/conductivity	Charge injection density	Biocompatibility	Flexibility	Advantages	Disadvantages	References
PPy	Template-assisted electro-deposition	Flat planar design	190 S/cm; doped with PSS; 19.84 S/cm	5 mC/cm^2^	Positive biocompatibility profile *in vivo*; increased neuron adhesion	Highly flexible	Flexibility; high electrical conductivity; biocompatible	Fragile mechanical properties; coating is thin; degradation possible	^76–78^
PEDOT:PSS	Crosslinked	3D printed micropillars	5.8 S/m	1.2–3.9 mC/cm^2^	Indirect and direct cytotoxic tests ISO 10993-5	Highly flexible	High electrical conductivity; transparency; biocompatible; neural stimulation demonstrated	Water soluble; long-term unstable	^64,79^
PPy/PSS layered with MWNTs	Layering and codeposition	N/A	30 S/cm	7.5 mC/cm^2^	Cell growth inhibition assay	Highly flexible	Electrochemically stable; high electrical conductivity	Requires process optimization; toxicity needs to be further verified	^78,80^
CNT	Low-pressure chemical vapor deposition	Vertically aligned pillars	1.8 × 10^7^ S/m	1.0 − 1.6 mC/cm^2^	Uncertain	Flexible	Highly electrically conductive; versatile	Surface modification required for biocompatibility; poor dispersion in composites	^70,71^
Porous graphene	Laser reduction	Coating	303 S/m	3.1 mC/cm^2^	Live/dead cell analysis	Flexible	Mechanically flexible; biocompatible; high electrical conductivity	May be fragile; requires surface modification to enhance hydrophilicity	^73,81,82^
Pt	Extrusion/drawing	Cylindrical and wire	9.6 × 10^6^ S/m	0.10–0.30 mC/cm^2^	MTT proliferation assay; smaller scar thickness	Rigid	Good mechanical properties; good biocompatibility; used extensively; chemically inert; high electrical conductivity	Cell death possible; possible corrosion; may undergo irreversible dissolution producing toxic by-product	^83,84^
	Chemical vapor deposition	Circle		295.07 μC/cm^2^					^83,85,86^
		Fractal		510.50 μC/cm^2^					
		Serpentine		318.82–359.53 μC/cm^2^					
Iridium oxide	Electrodeposition	Thin film	0.75 × 10^−3^ to 1.67 × 10^−3^ Ω cm	1.2 mC/cm^2^	Glial scar assay; neuron adhesion; MTT cell viability	Rigid	Good mechanical and electrical properties; high charge injection capacity	Over-pulsing can cause degradation; chronic usage may lead to inconsistency; less biocompatible compared to Pt	^86–89^
Titanium nitride	Sputtering	Thin film, coating	25 × 10^−6^ to 800 × 10^−6^ Ω cm	0.87 mC/cm^2^ @ 0.2 ms	MTT proliferation assay; live/dead cell analysis	Flexible coating	High surface areas; ease of fabrication	Potentially increased cell death; oxidation possible	^90–93^

3D, three-dimensional; CNT, carbon nanotube; MTT, 3-(4, 5-dimethylthiazolyl-2)-2, 5-diphenyltetrazolium bromide; MWNTs, multiwalled carbon nanotubes; PEDOT:PSS, poly(3,4-ethylenedioxythiophene) polystyrene sulfonate; PPy, polypyrrole; PPy/PSS, polypyrrole polystyrene sulfonate; Pt, platinum.

Intrinsically conducting polymers have found their application in neural stimulation *in vitro* due to their flexible mechanical nature, surface biocompatibility, and their tunable electrical conductivity. As an additional benefit these electrodes can provide varied EFs along the surface of the electrode unlike conventional metal electrodes. This was demonstrated using polypyrrole (PPy) with dodecyl benzene sulfonate as the dopant.^63^ The electrode contained regions with higher electrical conductivity and the neuronal and glial cells adhered more to those regions. Cross-linked poly(3,4-ethylenedioxythiophene) polystyrene sulfonate (xPEDOT:PSS) is another conductive polymer that stimulated NPC proliferation and differentiation *in vitro* with defined EF parameters.^64^ Furthermore, these intrinsically conducting polymers can be doped with other bioactive molecules to improve the microenvironment of damaged or diseased tissue, affording a combinatorial strategy to promote neural repair. One potential drawback is that these polymers may degrade in certain environments (e.g., higher pH at an injury site), which would require additional tuning of the parameters to support galvanotaxis.

**FIG. 2.**
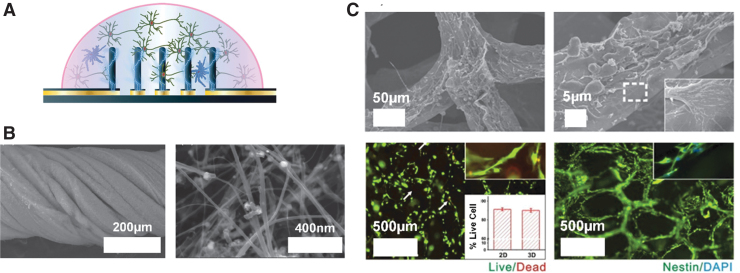
**(A)** PEDOT:PSS pillars were successfully 3D printed using a novel direct-write method, and electrical stimulation was applied to enhance NPC proliferation^65^; **(B)** CNT-based electrodes twisted into a rope morphology, which can be effectively utilized for neural stimulation. The graph on the right is the microscopy image of the synthesized CNTs at a higher magnification^68^; and **(C)** 3D graphene foam formation utilized as electrically conducting and biocompatible neural scaffolds for NPCs. The top two microscopy images show the structure of the graphene foams in detail and the region outlined with, while dashed line in the right image indicates the interaction between the cell and graphene foam surface. The bottom left image is a cell viability test with NPCs seeded on the graphene foam structure after 5 days of culturing, and the inset is the percentage live cell data (live cells—green, dead cells—red, and the arrows are indicating the dead cells). The bottom right image is a fluorescence image of NPC proliferation on the graphene foam surface (nestin for NPCs—green and DAPI for nuclei—blue).^69^ 3D, three-dimensional; CNTs, carbon nanotubes; NPC, neural precursor cell; PEDOT:PSS, poly(3,4-ethylenedioxythiophene) polystyrene sulfonate. Reproduced with permission. Copyright Wiley **(A, B)** and Nature **(C)**.

Another important consideration for the design and implementation of novel stimulating electrodes is the method of fabrication. Conducting polymers can be 3D printed (Fig. 2A) which supports the production of easily customizable shapes for electrodes.^65^ The ease and affordability of 3D printing have already been demonstrated with printed electrode connectors.^66^ A second method of fabrication is electrospinning. Electrospinning allows nonwoven fibrous composite electrodes to be fabricated by encapsulating conducting particles within biocompatible polymers while retaining its nanofibrous morphology. The conductive portions of the electrode are woven into the polymer, and this provides new surface geometry, further enabling different biocompatible polymers to be used. Yan et al. have electrospun hybrid fibrous electrode by integrating different concentrations of polyaniline tetramer with polycaprolactone and showed that NPCs were responsive to the stimulation and exhibited increased proliferation.^67^

Carbon nanotubes (CNTs) have unique properties in that they are structurally stable and very small, which are promising for neural electrode design. The smaller size leads to less tissue displacement upon implantation. Wang et al. fabricated CNT-based vertically aligned micropillars with small diameters of ∼50 μm for neural stimulation electrode arrays.^70^ Fabrication of CNTs is also advantageous as they can be twisted into a rope for increased contact area between the electrode and the tissue (Fig. 2B).^68^ Biocompatibility studies measuring the quantity of cytosolic enzyme lactate dehydrogenase, a marker of cell lysis, have shown that the CNT does not affect NPC survival. Most interestingly, the electrode design has unique surface properties that can impact cell interactions. Furthermore, functionalization of the CNT permits hydrophilic surfaces to form providing a safer electrode/tissue interface with high charge injection densities as a result of smaller interfacial resistance. While exciting, one concern is that CNTs may have biocompatibility issues *in vivo.* Therefore, simple surface modifications may be required to utilize CNTs to their full potential.^71^

Finally, graphene and graphene-oxide based electrodes are interesting platforms for electrical stimulation based on their inherent flexibility and biocompatibility.^72^ As shown in Figure 2C, Li et al. utilized graphene foams (GF) to provide an improved neural bioelectronic interface and stimulation scheme to regulate NPC migration and proliferation.^69^ It was further demonstrated that 3D GF could further enhance the NPC differentiation compared to its two-dimensional counter parts, if this is the desired outcome *in vivo.* The electrical conductivity of the GF structure decreased minimally in culture and provided control over the NPC migration and proliferation. Similar to the concerns for CNTs, graphene may still need surface modification to further improve biocompatibility *in vivo.*^73,74^

Composite neural electrode systems are constructed to combine the desired electrical properties for effective and safe stimulation, with a supporting mechanical scaffold potentially for cell adhesion. Recently, Fu et al. have created a poly(l-lactic-co-glycolic acid) (PLGA)/graphene oxide (GO) composite film to be used as neural stimulation platform.^75^ The PLGA/GO system was sufficient to promote stem cell proliferation and neurite elongation in differentiated neurons making it a promising material for future *in vivo* studies.

Overall, there exist a variety of flexible and biocompatible materials that are capable of stimulating NPCs and modifying their survival, proliferation kinetics, and differentiation profiles *in vitro*. The challenge lies in translating them to *in vivo* electrodes that will minimize damage to the microenvironment and provide electrical stimulation sufficient to promote migration to the target locations.

## Surface and Geometry: Is the Solution Shaping Up?

The surface and geometry of implanted electrodes can play important roles in modulating galvanotaxis for neurostimulation since electrode surface features and overall geometry impact on electrode parameters such as electrical impedance, charge injection capacity, and stimulation efficiency.^94^ Surface and geometry should be optimized to maximize the clinical effectiveness while minimizing the risks associated with electrical stimulation of the brain or tissue trauma during electrode implantation. In this section, the effect of surface morphology and geometrical features on neurostimulation is outlined.

**FIG. 3.**
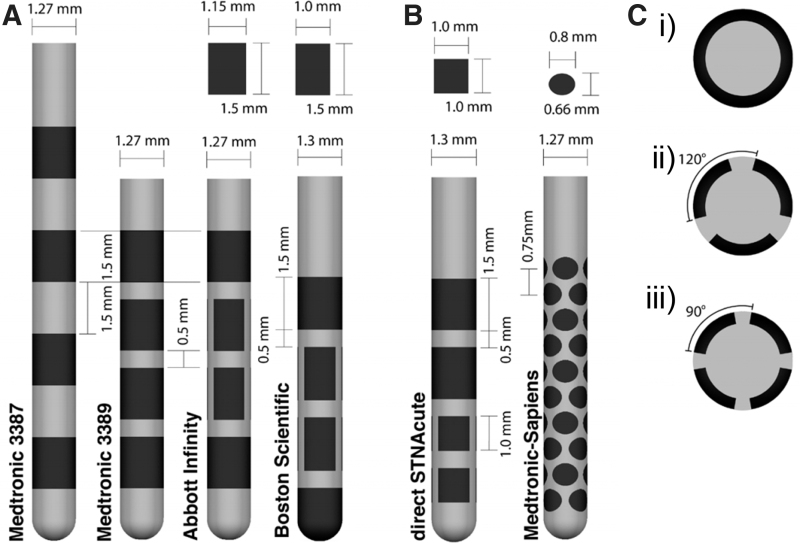
**(A)** FDA-approved commercial cylindrical DBS electrodes are mainly simple rectangular shapes with quadripolar arrangements. Featured here: Medtronic 3387/3389, the Abbott Infinity lead and the Boston Scientific lead. For the Abbott Infinity and Boston Scientific lead, the dimensions of the three contacts around the circumference of the middle two rows can be found above. **(B)** Different geometries and arrangements are featured in the direct STNAcute and Medtronic-Sapiens lead that have been implanted in human patients for testing but not approved by the FDA. **(C)** Cross-sections of the DBS electrodes: **(C.i)** Medtronic 3387/3389 and other cylindrical contacts on directional leads. **(C.ii)** Three segments on the Abbott directional lead, Boston Scientific directional lead, and the direct STNAcute. **(C.iii)** Four segments on the Medtronic-Sapiens. Dark regions on the implants correspond to the active electrode placements, while the gray regions indicate the supporting structures. DBS, deep brain stimulation. Figure reproduced under the terms of the Creative Commons Attribution 3.0 License.^103^

**FIG. 4.**
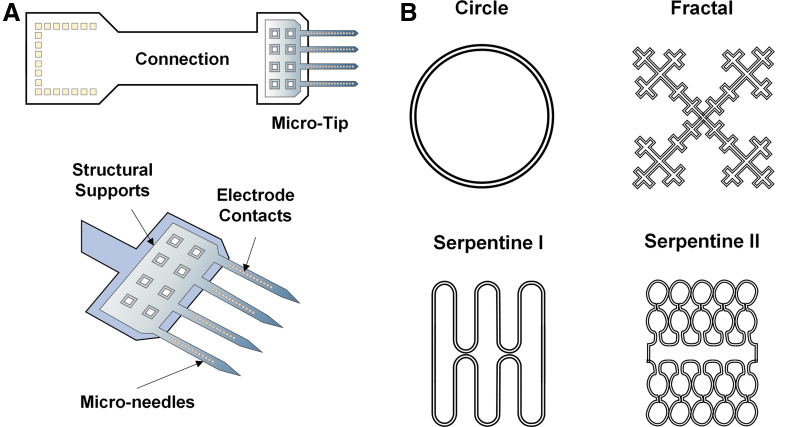
**(A)** Gold microelectrode probes with an array of 32 channels. Surface roughness was increased by electroplating iridium on gold electrode sites to enhance the capacitive characteristics.^96^ **(B)** Geometries with identical surface areas but different perimeters. Reproduced with permission. Copyright Nature **(B)**.

The effect of surface features on the performance of the commercial DBS electrode designs (Fig. 3) has been explored with the understanding that electrical properties of electrodes could be improved by considering surface morphology, shape, and size optimization. For instance, Yamagiwa et al. compared porous and flat metal electrodes made of the same material (titanium nitride and iridium oxide) and showed that rough electrodes have lower impedance and higher charge-injection capacity than flat ones, a beneficial property for stimulation electrodes.^95^ In another study, the surface roughness was increased to enhance capacitive characteristics by electroplating of iridium on gold electrode microarrays (Fig. 4A).^96^ There are a number of benefits to porous electrode surfaces; however, one caveat is the possibility of biofilm formation which can increase the risk of infection. Surface modifications such as nanotechnologies to prevent biofilm formation are worthy of consideration.^97,98^

Lee and colleagues studied the role of geometries on electrode electrical properties by fabricating fractal and serpentine-shaped platinum electrodes and compared them with traditional circular electrodes (Fig. 4B). Under the same area, Serpentine II had the highest perimeter and thus exhibited the greatest charge storage capacity and total delivered current. In addition, the fractal-shaped electrode exhibited an outstanding charge injection capacity and potential penetration capability due to greater Faradaic and non-Faradaic electrochemical processes.^99^ Fractal shapes have also been shown to improve the energy efficiency requiring only 78% of the original input power needed to maintain the same level of neurostimulation.^100^ Although noncircular electrodes provide better electrical performance, risks of damage due to sharp edges have not been examined *in vivo*. Nevertheless, these geometry patterns can be incorporated to traditional cylindrical electrodes and flexible electrode microarrays to promote effective neurostimulation while maintaining their signal quality and biocompatibility.

Reduction of electrode lead size from millimeters to micrometers effectively decreases the volume of the implant, which will decrease mechanical strain at the implantation site and reduce the inflammatory response. For instance, polypropylene fibers with micrometer-sized diameter exhibited significantly smaller macrophage density compared to larger diameter fibers. One caveat is the greater risk of breakage with the smaller diameter fibers.^101^ These results are consistent with another study, which compared two different sizes of stainless-steel electrode coated with poly-glycolic acid at 4 weeks postimplantation. Smaller diameter (12 μm) electrodes resulted in significantly less glial scar formation than the larger diameter (25 μm) electrode^102^ supporting the conclusion that size optimization should carefully be considered for electrode design.

Overall, theoretical and experimental studies indicate that the geometry has a tremendous effect on neurostimulation. However, none have looked at the effects of these changes on electrosensitive NPCs in the brain. In the future, these studies will be critical for exploiting EFs as a means to promote neural repair.

## A Clinical Perspective: A Stimulating Future Outlook

Together, the developments discussed above could enable the use of EFs for applications that go well beyond the circuit modulation paradigm currently targeted by DBS protocols. Three clinical applications are of particular interest: galvanotaxis, neuroprotection, and spatiotemporal control of delivery of therapeutics, including drugs and viral-mediated gene therapies. These clinical applications could provide new therapies for the many individuals that are living with neurological disorders. Finally, we highlight two technological advances that can be used for these clinical applications: electrode modifications and EF modeling.

### Galvanotaxis

The success of stem cell-based clinical trials in stroke or spinal cord injury has been hampered, in part, by the realization that NPCs need an appropriate environment to reach the diseased target, to differentiate into appropriate cell types, and organize into functional networks.^104^ Promising work has shown that murine NPC survival, migration, and differentiation can be modified by EF application. Indeed, human trials using scaffolds have been conducted to address these issues^105,106^; however, a major limitation in their utility is that artificial channels cannot be adjusted after implantation. If properly controlled, galvanotaxis could provide a means to direct NPC migration through more permissive mediums, such as hydrogels, or to enhance NPC migration along endogenous migration paths such as the corpus callosum (Fig. 1B). Furthermore, dynamic adjustment of the EF as the target tissue is being regenerated could allow for improved neuroplasticity, including synaptic reconstitution and network restoration.

### Neuroprotection

Although we primarily discussed the potential of electrical stimulation to promote NPC migration for tissue regeneration, evidence is accumulating that DBS may have direct neuroprotective, disease-modifying effects outside of migratory responses.^107^ A number of reports of subthalamic nucleus DBS for Parkinson's Disease reveal a trend toward motor score stabilization or improvement when off-medication/off-stimulation in chronically stimulated patients.^108^ While the neurological substrate of this stabilization is yet to be determined in humans, animal studies have shown that chronic subthalamic nucleus DBS protected the dopaminergic neurons from cell toxicity in Parkinson's Disease in a number of animal models.^109–113^ Another example is Alzheimer's disease where DBS of the fornix leads to a reduction in the rate of hippocampal atrophy.^114^ The mechanism of action in both diseases is unknown, but is consistent with a reduction of excitotoxicity from hyperactive glutamatergic projections^111,115,116^ or the induction of neurotrophic factor release.^117–119^ In neurodegenerative diseases where specific structures are preferentially affected, targeted neuroprotective stimulation in the early stages of the disease could halt disease propagation across the affected neural network.

### Spatiotemporal control of other therapeutics

While EFs can modulate cellular function with exquisite spatial and temporal resolution, they are hardly tissue selective. Targeting dopaminergic neurons without affecting colocalized cholinergic projections, for example, cannot be reliably achieved with current DBS programming paradigms. A promising approach to obtain tissue selectivity is through the use of drugs or viral vectors targeting tissue-specific receptors on the cells. Indeed, using electroresponsive drugs or viral mediated gene therapies could provide the best of both worlds by enabling EFs to exert spatiotemporal control over the activation of tissue-selective drugs or gene-specific promoters. This approach is currently being used with considerable success in optogenetics studies,^120^ although light penetration through tissue limits the size and location of optogenetic targets.^121^ It is exciting to speculate that “electrogenetics” could circumvent these limitations and, indeed, proof of concept studies are underway.^122^

### Adaptable electrodes

Modified approaches to enhance neural repair using shape-memory polymers, for example, could provide adaptive control over the NPC migration path as cells migrate enabling the “best” cell migration performance to be achieved. Customizable electrodes could adapt to the patient-specific microenvironments and even change shape to create a better fit to ensure the EFs are effectively applied.^123^ Additional concepts of adaptable electrodes also include tailorable electrical conductivity and mechanical flexibility based on the *in vivo* environment, allowing the most effective electrical stimulation to be applied.^123,124^ Furthermore, it is possible to create electrodes that undergo harmless degradation and residual absorption once electrical stimulation is completed, which is an exciting concept that would effectively eliminate the need for further surgery to remove the electrode after tissue regeneration.^125^

### EF modeling

Finally, a better understanding of the EF distribution *in vivo* is necessary to better predict outcomes related to these novel therapeutic interventions. Multiphysics modeling tools using measured electrical conductivities of the cerebral cortex, corpus callosum, and the lateral ventricles (e.g.) would facilitate an understanding of the EF lines in the brain *in vivo*.^126^ Customization of these conductivities and modeling could be performed to consider hydration status, disease progression, and even mental state of the participants using close-looped systems measuring local field potentials to adjust the state of the stimulation accordingly.^127^ Using these analyses, the optimal electrode configuration and customized stimulation paradigms can be predicted to provide the best stimulation performance. The directionality and strength of the applied EF *in vivo* and the electrodes' interactions with the adjacent neural tissues will offer valuable insights in the fundamental mechanisms underlying the results of the stimulation of these new clinical applications.

## Conclusion

We have highlighted the promise of EF application for the goal of improving neuroplasticity and promoting neural repair. The presence of endogenous NPCs has generated excitement about their use in neural regeneration strategies. We propose that harnessing their electrosensitive properties is a novel approach to treat the injured or diseased CNS. NPCs respond to electrical stimulation through migration, proliferation, and differentiation. Controlling their behavior requires a sound knowledge of the impact of EFs on brain tissue, brain pathology, and how the brain perceives the EF. The design and manufacturing of stimulating electrodes specifically created for the purpose of neural repair are underway. To enable more effective future neural stimulation for tissue repair, next generation materials and optimized geometries will also need to be considered. The future is just around the corner.
